# Supplementary material for: Patient factors affecting successful linkage to treatment in a cervical cancer prevention program in Kenya: A prospective cohort study
Source: PLoS One. 2019 Sep 18;14(9):e0222750. doi: 10.1371/journal.pone.0222750 (PMC6750649; doi:10.1371/journal.pone.0222750)
Supplement: Attachment — Submitted filename: CP Kenya PLOS ONE response to reviewers.docx [file pone.0222750.s002.docx]

**Manuscript: PONE-D-19-17741**

We thank the editor and reviewers for your careful reading of our manuscript and for your thoughtful comments. We have incorporated your suggestions into the updated version of our manuscript as detailed below. Please note that line numbers in our responses below refer to the tracked version of the revised manuscript.

**Responses to comments from the editor:**

*1. Please ensure that your manuscript meets PLOS ONE's style requirements, including those for file naming. The PLOS ONE style templates can be found at*

[*http://www.journals.plos.org/plosone/s/file?id=wjVg/PLOSOne_formatting_sample_main_body.pdf*](https://urldefense.proofpoint.com/v2/url?u=http-3A__www.journals.plos.org_plosone_s_file-3Fid-3DwjVg_PLOSOne-5Fformatting-5Fsample-5Fmain-5Fbody.pdf&d=DwQGaQ&c=imBPVzF25OnBgGmVOlcsiEgHoG1i6YHLR0Sj_gZ4adc&r=6sAo_faMnl18SymMIA0IhoOiK1Owiy7olSBVGdttjBw&m=tkPEVJqwYq8Qq0CbpJkXzQeMf5vg3oMv1WUfvXagnmY&s=8_Iva6jEYG6M7gFSdJoQJJwOhhbd63ZOOBeVfykWLyw&e=) *and*[*http://www.journals.plos.org/plosone/s/file?id=ba62/PLOSOne_formatting_sample_title_authors_affiliations.pdf*](http://www.journals.plos.org/plosone/s/file?id=ba62/PLOSOne_formatting_sample_title_authors_affiliations.pdf)

We have reviewed PLOS ONE’s style requirements to ensure that our manuscript is compliant. We made changes in the punctuation around the in-text citations.

*2. In your Methods section, please provide additional information about the participant recruitment method, including a statement as to whether your sample can be considered representative of a larger population, and a description of how participants were recruited.*

Thank you for this suggestion. We have added the requested information to the Methods section (lines 103-8).

*3. We note that you have indicated that data from this study are available upon request. PLOS only allows data to be available upon request if there are legal or ethical restrictions on sharing data publicly. For information on unacceptable data access restrictions, please see* [*http://journals.plos.org/plosone/s/data-availability#loc-unacceptable-data-access-restrictions*](https://urldefense.proofpoint.com/v2/url?u=http-3A__journals.plos.org_plosone_s_data-2Davailability-23loc-2Dunacceptable-2Ddata-2Daccess-2Drestrictions&d=DwQGaQ&c=imBPVzF25OnBgGmVOlcsiEgHoG1i6YHLR0Sj_gZ4adc&r=6sAo_faMnl18SymMIA0IhoOiK1Owiy7olSBVGdttjBw&m=tkPEVJqwYq8Qq0CbpJkXzQeMf5vg3oMv1WUfvXagnmY&s=_HnNskm0Fp4mbdX3h-T-qrY486_p2zh31VgewRuCkR4&e=)*.*

*In your revised cover letter, please address the following prompts:*

*a) If there are ethical or legal restrictions on sharing a de-identified data set, please explain them in detail (e.g., data contain potentially identifying or sensitive patient information) and who has imposed them (e.g., an ethics committee). Please also provide contact information for a data access committee, ethics committee, or other institutional body to which data requests may be sent.*

*b) If there are no restrictions, please upload the minimal anonymized data set necessary to replicate your study findings as either Supporting Information files or to a stable, public repository and provide us with the relevant URLs, DOIs, or accession numbers. Please see* [*http://www.bmj.com/content/340/bmj.c181.long*](https://urldefense.proofpoint.com/v2/url?u=http-3A__www.bmj.com_content_340_bmj.c181.long&d=DwQGaQ&c=imBPVzF25OnBgGmVOlcsiEgHoG1i6YHLR0Sj_gZ4adc&r=6sAo_faMnl18SymMIA0IhoOiK1Owiy7olSBVGdttjBw&m=tkPEVJqwYq8Qq0CbpJkXzQeMf5vg3oMv1WUfvXagnmY&s=SVLxuLLfsEZcj9eTHwovkARU3Ns8HBin4WvUw8tWcps&e=) *for guidelines on how to de-identify and prepare clinical data for publication. For a list of acceptable repositories, please see* [*http://journals.plos.org/plosone/s/data-availability#loc-recommended-repositories*](https://urldefense.proofpoint.com/v2/url?u=http-3A__journals.plos.org_plosone_s_data-2Davailability-23loc-2Drecommended-2Drepositories&d=DwQGaQ&c=imBPVzF25OnBgGmVOlcsiEgHoG1i6YHLR0Sj_gZ4adc&r=6sAo_faMnl18SymMIA0IhoOiK1Owiy7olSBVGdttjBw&m=tkPEVJqwYq8Qq0CbpJkXzQeMf5vg3oMv1WUfvXagnmY&s=NlYbakZ5zGWCPmAVqLZ-IYznjC6DLGwfw14h1s5fiWk&e=)*.*

*We will update your Data Availability statement on your behalf to reflect the information you provide.*

There are no ethical restrictions to sharing a de-identified dataset. We now include this dataset as Supporting Information file “S2 Dataset.”

**Responses to comments from the reviewers:**

*Reviewer #1: Line 102- Women with cervical lesions not amenable to cryotherapy or
103 suspicious for cancer were referred to a gynecologist. What was the distance women had to travel to see a gynecologist and what was the waiting time?*

These are excellent questions. Women were referred to a gynecologist at Migori County Referral Hospital, which is in the capital of Migori County. We have added this information to our Methods section (lines 122-3). The geodetic distance to this site from the homes of women in the study population ranges from 4 to 40 km, with a median of 29 km. We do not have information on the waiting time; however, the supervising gynecologist was a consultant on the study and worked to facilitate care for all referred women in a timely fashion.

*Line 112- The study population for the main trial were women in Migori County who were eligible
113 for cervical cancer screening based on the Kenya Ministry of Health’s guidelines: 25-65 years old with an intact uterus and cervix. – add reference*

We have added a reference and removed the statement about “an intact uterus and cervix” after clarifying against the reference (lines 136-7).

*Reviewer #2: Reviewer Comments
“Patient factors affecting successful linkage to treatment in a cervical cancer prevention program in Kenya: a prospective cohort study”

General comment
• The manuscript is well written however there are some comments in each of the section that could improve the manuscript.
Abstract
• In the results section provide direction of association for the significant variables whether positive or negative*

This is a good suggestion. We have updated the abstract accordingly (lines 39-42).

*Introduction
More background need to be provided on the linkage rate after screening in the region and in the whole country in general. This is not clear before assessing factors what is the burden.*

Since this is the first protocol in Kenya to incorporate hrHPV testing as part of screening through government health facilities, there is no linkage to treatment rate to which to compare outside of this study. However, in Phase 1 of this study, which employed standard linkage to treatment, less than 50% of women were successfully linked. We have included this data in the Methods section (lines 89-90).

*Methodology
Were the participants counseled prior or after testing?*

The participants were counseled prior to testing. We have provided clarification about this counseling in lines 108-9.

*In the methods it is stated that this current manuscript focuses on the group that was exposed to enhanced linkage strategy that included transport facilitation for a limited number of participants. How many among the one studied had facilitated transportation? How was this handled in the analysis.*

We clarified with the study coordinator, and there actually was no transport facilitation for women in Phase 2 of the study, which is the phase of interest for this research. We apologize for this error and have removed this statement from the manuscript (line 94).

*Results
• The variable Missed work to come to the CHC for screening was this for women who were employed or for all women*

This variable applied to all women. We think there is something about the decision and ability to miss work that separates women who missed work from those who either work inside the home or work outside the home and didn’t miss work. In preparing our response to this question, we realized that the frequencies in Table 1 for this variable were incorrect; they are now accurate.

*• How were the variables “Told by partner, family member, or someone else important to come to the CHC” and “Told by partner, family member, or someone else important not to come to the CHC” phrased since one is the negative of the other.*

The questions were worded to participants at the CHCs as follows: “Did your partner, family member, or anyone else important to you tell you to come?” and “Did your partner, family member, or anyone else important to you tell you *not* to come?” During training and piloting of the surveys, we had the research assistants stress the difference between the two questions.

*• “Women with a college education or beyond were not significantly more likely to present for treatment than women in the least educated category (OR 2.17, 95% CI 0.65-7.26), with a wide confidence interval in the setting of very few women having a college education or more”. Why not consider collapsing the groups?*

It would certainly be reasonable to combine the groups “at least some secondary” and “college or beyond”; however, we chose to keep them separate to provide more detailed data on the distribution of education levels in the study population.

*• Was multicollinearity before the adjusted odds ratio were obtained.*

Yes, we did test for multicollinearity among the variables that we used in the multivariable logistic regression models. The variance inflation factor (VIN) for all variables was <2, indicating that collinearity is not a concern.

*Data Availability
Indicated that the data is available but no link to the data*

We now attach the de-identified data as a Supporting Information file.
