# Supplementary material for: Patient factors affecting successful linkage to treatment in a cervical cancer prevention program in Kenya: A prospective cohort study
Source: PLoS One. 2019 Sep 18;14(9):e0222750. doi: 10.1371/journal.pone.0222750 (PMC6750649; doi:10.1371/journal.pone.0222750)
Supplement: Attachment — Submitted filename: Reviewer_comments_1.docx [file pone.0222750.s003.docx]

**Reviewer comments**

1. The variable Missed work to come to the CHC for screening was this for women who were employed or for all women
   Response: This variable applied to all women. We think there is something about the decision and ability to miss work that separates women who missed work from those who either work inside the home or work outside the home and didn’t miss work. In preparing our response to this question, we realized that the frequencies in Table 1 for this variable were incorrect; they are now accurate.

**Comment**: How many among those working outside home missed work to come to CCC. Since the ones at home might not have considered themselves to have missed work. This all depends on how the questions was phrased and from the frequencies the number missing work is lower than the number working outside home.

2. “Women with a college education or beyond were not significantly more likely to present for treatment than women in the least educated category (OR 2.17, 95% CI 0.65-7.26), with a wide confidence interval in the setting of very few women having a college education or more”. Why not consider collapsing the groups?
   Response: It would certainly be reasonable to combine the groups “at least some secondary” and “college or beyond”; however, we chose to keep them separate to provide more detailed data on the distribution of education levels in the study population.

**Comment**: Due to the small numbers in the College group why not combine them with the secondary to gain more power. Then we can be able to make the conclusion that education is a factor currently its only between secondary and primary.
