# Supplementary material for: Patient factors affecting successful linkage to treatment in a cervical cancer prevention program in Kenya: A prospective cohort study
Source: PLoS One. 2019 Sep 18;14(9):e0222750. doi: 10.1371/journal.pone.0222750 (PMC6750649; doi:10.1371/journal.pone.0222750)
Supplement: Attachment — Submitted filename: CP Kenya PLOS ONE response to reviewers v.2.docx [file pone.0222750.s004.docx]

**Manuscript: PONE-D-19-17741**

Response: We thank the editor and reviewers for your careful reading of our manuscript and for your thoughtful comments. We have incorporated your suggestions into the updated version of our manuscript as detailed below. Please note that line numbers in our responses below refer to the tracked version of the revised manuscript.

**Responses to comments from Reviewer 2:**

1. The variable Missed work to come to the CHC for screening was this for women who were employed or for all women
   Response: This variable applied to all women. We think there is something about the decision and ability to miss work that separates women who missed work from those who either work inside the home or work outside the home and didn’t miss work. In preparing our response to this question, we realized that the frequencies in Table 1 for this variable were incorrect; they are now accurate.

**Comment**: How many among those working outside home missed work to come to CCC. Since the ones at home might not have considered themselves to have missed work. This all depends on how the questions was phrased and from the frequencies the number missing work is lower than the number working outside home.

Response: The questions were phrased as, “Are you currently working outside of the home?” and “Did you have to miss work to come to this campaign?” Six percent of women who do not work outside the home reported missing work to come to the CHC, and 57% of women who work outside the home reported doing so. We have added this data to the text of the manuscript (lines 177-9). The 2x2 table is below.

|  | Missed work | Did NOT miss work |
| --- | --- | --- |
| Work outside the home | 165 | 126 |
| Do NOT work outside the home | 12 | 202 |

When the analysis was limited to the 291 women who work outside the home, the association between missing work to come to the CHC and presenting for treatment remained significantly significant (2.01, 95% confidence interval 1.26-3.22).

2. “Women with a college education or beyond were not significantly more likely to present for treatment than women in the least educated category (OR 2.17, 95% CI 0.65-7.26), with a wide confidence interval in the setting of very few women having a college education or more”. Why not consider collapsing the groups?
   Response: It would certainly be reasonable to combine the groups “at least some secondary” and “college or beyond”; however, we chose to keep them separate to provide more detailed data on the distribution of education levels in the study population.

**Comment**: Due to the small numbers in the College group why not combine them with the secondary to gain more power. Then we can be able to make the conclusion that education is a factor currently its only between secondary and primary.

Response: We have combined the college group with the secondary education group and updated the statistical analysis to reflect this change.
